# Encapsulated Whole Bone Marrow Cells Improve Survival in Wistar Rats after 90% Partial Hepatectomy

**DOI:** 10.1155/2016/4831524

**Published:** 2015-11-16

**Authors:** Carolina Uribe-Cruz, Carlos Oscar Kieling, Mónica Luján López, Alessandro Osvaldt, Gustavo Ochs de Muñoz, Themis Reverbel da Silveira, Roberto Giugliani, Ursula Matte

**Affiliations:** ^1^Gene Therapy Center, Hospital de Clínicas de Porto Alegre, Ramiro Barcelos 2350, 90035-903 Porto Alegre, RS, Brazil; ^2^Post-Graduation Program on Genetics and Molecular Biology, Federal University of Rio Grande do Sul, Porto Alegre, RS, Brazil; ^3^Experimental Hepatology and Gastroenterology Laboratory, Experimental Research Center, Hospital de Clínicas de Porto Alegre, Ramiro Barcelos 2350, 90035-903 Porto Alegre, RS, Brazil; ^4^Post-Graduation Program in Science of Gastroenterology and Hepatology, Federal University of Rio Grande do Sul, Porto Alegre, RS, Brazil; ^5^Post-Graduation Program in Surgery, Federal University of Rio Grande do Sul, Porto Alegre, RS, Brazil; ^6^Post-Graduation Program in Adolescent and Child Health, Federal University of Rio Grande do Sul, Porto Alegre, RS, Brazil

## Abstract

*Background and Aims*. The use of bone marrow cells has been suggested as an alternative treatment for acute liver failure. In this study, we investigate the effect of encapsulated whole bone marrow cells in a liver failure model. *Methods*. Encapsulated cells or empty capsules were implanted in rats submitted to 90% partial hepatectomy. The survival rate was assessed. Another group was euthanized at 6, 12, 24, 48, and 72 hours after hepatectomy to study expression of cytokines and growth factors. *Results*. Whole bone marrow group showed a higher than 10 days survival rate compared to empty capsules group. Gene expression related to early phase of liver regeneration at 6 hours after hepatectomy was decreased in encapsulated cells group, whereas genes related to regeneration were increased at 12, 24, and 48 hours. Whole bone marrow group showed lower regeneration rate at 72 hours and higher expression and activity of caspase 3. In contrast, lysosomal-*β*-glucuronidase activity was elevated in empty capsules group. *Conclusions*. The results show that encapsulated whole bone marrow cells reduce the expression of genes involved in liver regeneration and increase those responsible for ending hepatocyte division. In addition, these cells favor apoptotic cell death and decrease necrosis, thus increasing survival.

## 1. Introduction

Acute liver failure (ALF) is characterized by the sudden loss of liver function that results in jaundice, coagulopathy, and hepatic encephalopathy in a previously healthy individual. If not treated it can lead to renal and multiple organ failure, coma, and death [1]. Orthotropic liver transplantation is the treatment of choice for ALF although the lack of a suitable donor in a short period of time can limit the success of this therapy [2]. In addition to that, the lifelong use of immunosuppressant after the transplant possesses side effects in the short and long term [3, 4]. These observations and the high costs of the procedure and its complications have led to the search for alternative approaches to ALF that do not include liver transplant.

The use of bone marrow-derived cells in regenerative medicine has grown in the past years. Their efficacy has been shown in animal models of both chronic [5, 6] and acute liver disease [7–9]. They present several advantages when compared to hepatocytes as they are readily available and can be expanded* in vivo* or* in vitro* [10]. In addition, the use of autologous cells would eliminate the need for immunosuppressants [11]. In animal models, heterologous transplantation of mesenchymal stem cells [12] or encapsulated bone marrow cells [13] is also performed without immunosuppressants. However the mechanisms by which these cells exert their beneficial effect on liver regeneration are not completely well understood. They may involve an increase in the number of hepatocytes by either transdifferentiation, fusion, and/or the secretion of paracrine factors that stimulate cell division, inhibit apoptosis, or modulate local and systemic inflammatory state [10, 14].

Several proinflammatory factors are involved in the early phase of liver regeneration. After partial hepatectomy, the increased amounts of enteric lipopolysaccharides (LPS) that bind to Tlr-4 (toll like receptor 4) on Kupffer cells activate the MYD88 (myeloid differentiation factor) pathway and trigger the activation of Nf*κ*-B (nuclear factor kappa B) and the release of Tnf-*α* (tumor necrosis factor-*α*) and Il-6 (interleukin-6) [15]. Il-6 plays a key role in liver regeneration, activating acute phase genes and priming hepatocytes to growth factors [16, 17]. Hgf (hepatocyte growth factor) then stimulates hepatocytes to pass from G0 to G1, thus initiating the cell cycle [18, 19]. The increase in molecules such as Socs3 (suppressor of cytokine signaling 3) and Tgf-*β*1 (transforming growth factor-beta) contributes to the decrease in stimulating factors and the halt of liver regeneration [18, 20].

After partial hepatectomy, there is a complex remodeling of the liver tissue with a transient disruption of the lobular architecture [21]. Agglomerates of poorly vascularized hepatocytes are formed in the periportal area before invasion of sinusoidal cells [20, 22]. Some authors have suggested that at the early stages of liver regeneration a very fine tuning in the rate of proliferation of parenchymal and nonparenchymal cells is needed. Ninomiya et al. [22] showed that a slowed hepatocyte regeneration rate increased the survival in a model of 90% partial hepatectomy.

Our goal was to investigate the paracrine effects of bone marrow cells and the mechanisms by which they increase survival in a rat model of 90% partial hepatectomy.

## 2. Methods

### 2.1. Animals

Two-month-old male outbred Wistar rats, weighing 310.5 ± 33 g, were housed under controlled temperature (between 18 and 22°C) in light-dark cycles of 12 h with free access to water and standard chow at the Experimental Animal Unit at Hospital de Clínicas de Porto Alegre (HCPA). Handling, care, and processing of animals were carried out according to regulations approved by our local ethics committee (protocol number 10-0062) and complied with the National Guidelines on Animal Care.

### 2.2. Experimental Design

Animals were submitted to 90% partial hepatectomy (90% PH) and randomly divided in two groups. Treated group received encapsulated whole bone marrow cells (WBM, *n* = 11) and control group (*n* = 15) received empty capsules (EC). Survival was observed for up to 10 days after 90% PH. An additional set of animals from both groups was sacrificed at 6, 12, 24, 48, and 72 hours after 90% PH (*n* = 6/group/time point) to evaluate the early effects of treatments.

### 2.3. Isolation of Whole Bone Marrow Cells and Encapsulation

Thirty-three animals without liver injury were used as donors of WBM cells. In a sterile environment, the femurs and tibias were isolated and WBM from each bone was flushed with 3 mL complete medium: DMEM (Dulbecco's Modified Eagle Medium, LGC, Brazil) supplemented with 10% fetal bovine serum (Gibco, USA), 1% penicillin/streptomycin (Gibco, USA). Cell viability was determined by Trypan's Blue exclusion.

Cell encapsulation was performed according to our laboratory protocol, previously described [23]. Briefly, WBM cells were mixed with 1.5% sodium alginate (Sigma-Aldrich, USA) in complete medium and extruded through an Encapsulation Unit type J1 (Nisco, Switzerland), attached to JMS Syringe Pump. Droplets were sheared off with an air flow of 5 L/min delivered to the tip of a 27 G needle and the rate of infusion was 40 mL/h. The droplets fell into a bath of 125 mM CaCl_2_ and ionically cross-linked with Ca_2_^+^ to form solid spherical hydrogel beads containing embedded WBM cells. For control group, empty capsules were produced using the same approach, although without cells. The resulting capsules were maintained under normal cell culture conditions with complete medium at 37°C and 5% CO_2_ for 24 h prior to transplantation.

### 2.4. Surgical Procedure and Capsules Transplantation

Ninety percent hepatectomy was performed by a single operator as described by Gaub and Iversen [24]. In brief, the left lateral (30%), left median (40%), and right superior lobes (20%) were removed, leaving only the caudate lobes. Hepatectomy was carried out under isoflurane (Forane, Abbott SA, Argentina) anesthesia [25]. Immediately after 90% PH and before complete suture, microcapsules (containing 3 × 10^7^ WBM cells [26] or empty) were placed into the peritoneal cavity and glucose was supplemented i.p. (5% of body weight). Postoperatively, animals were given i.p. glucose (5% of body weight) until day seven and received 20% glucose in their drinking water and standard chow* ad libitum*.

### 2.5. Euthanasia

Euthanasia was performed in CO_2_ chambers. To evaluate survival, the animals were euthanized 10 days after 90% HP. To evaluate the early effects of treatments the animals were euthanized 6, 12, 24, 48, and 72 h after 90% HP and immediately blood was collected, the liver was removed and weighed, and part was flash frozen in liquid nitrogen or set at paraffin.

### 2.6. Quantitative Real-Time PCR

Total RNA was extracted from 50 mg of liver tissue using TRIzol reagent (Invitrogen, USA) according to the manufacturer's instructions. Two micrograms of RNA was reverse-transcribed using High Capacity cDNA Reverse Transcription Kit (Life Technologies, USA). Gene expression was measured using TaqMan assays (Life Technologies, USA) for genes involved in hepatic regeneration (Table 1). The percentage of a test RNA to that of *β*-actin was calculated by subtracting the cycle to reach the threshold (CT) for a gene from the CT for a separate assay using *β*-actin assay to determine the ΔCT and the following formula: percent *β*-actin = (100) ×  2^ΔCT^ [27]. The percent *β*-actin for hepatectomized animals was divided by the percent *β*-actin in normal animals to determine the ratio of the gene in both treatments after 90% PH to normal rats. Livers of animals without injury were used as calibrator group (*n* = 5).

### 2.7. Liver Regeneration Rate

The liver regeneration rate was calculated as follows: liver regeneration rate (%) = 100 × [*C* − (*A* − *B*)]/*A*, where *A* is the estimated liver weight before PH, *B* is the excised liver weight at the time of PH, and *C* is the weight of the regenerated liver at the time of sacrifice [28].

### 2.8. Histology

Paraffin-embedded liver specimens were cut in 4 *μ*m sections and stained with hematoxylin and eosin (H-E). To assess the rate of hepatocyte proliferation, the number of hepatocytes undergoing mitosis was counted in 10 high-power fields (HPF) in 72 hs after HP (mitotic index) [29].

To determine the number and the size of parenchymal cells per slide, the hepatocytes nuclei were counted and internuclear distance was measured in 5 HPF using Cell Imaging Software for Life Science Microscopy (Olympus) at 72 h after HP.

### 2.9. Enzyme Assays

Fluorimetric caspase activity (Sigma-Aldrich, USA) assays were performed according to manufacturer's instruction. Briefly, approximately 100 *μ*g of liver was placed in an opaque 96-well plate and 200 *μ*L of mixture reaction solution (containing acetyl-Asp-Glu-Val-Asp-7-amido-4-methylcoumarin) was added in each well. The plate was incubated in dark at 25°C and every 10 minutes the fluorescence was read at 360 nm of excitation and 460 of emission. Caspase activity was normalized by protein.

For lysosomal-*β*-glucuronidase (Gusb) measurement, livers were homogenized in PBS buffer with proteases inhibitor cocktail 1%. Assays were performed using the chromogenic substrates 4-methylumbelliferyl-*β*-L-glucuronide (Sigma-Aldrich) at pH 4.5. One unit of enzyme activity converts 1 nmol of substrate to product per hour at 37°C.

### 2.10. Statistical Analysis

Results were expressed as means ± standard deviation (SD) or medians when required. Statistical differences were assessed by Student's *t*-test and for nonparametric variables Mann-Whitney test was used. The survival rate was analyzed by Kaplan-Meier curve. The comparison of survival rates in different groups was tested by the log rank test. *P* values less than .05 were considered statistically significant.

## 3. Results

### 3.1. Survival Rate

**Figure 1:**
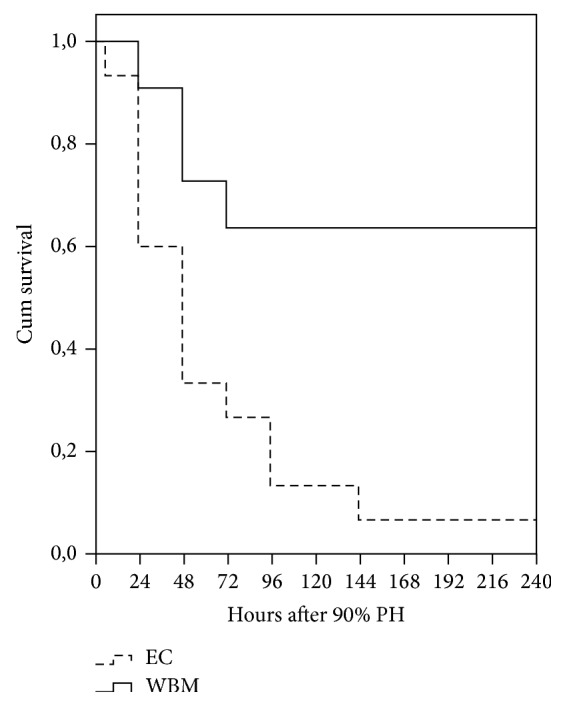
Spontaneous survival according to therapeutic regimen in rats after 90% partial hepatectomy (PH). EC: empty capsules, WBM: whole bone marrow, and Cum: cumulative. Log rank = .002.

Overall survival rate was observed during 10 days after hepatectomy. The survival rate was higher for the WBM group (63.6%) than for EC group (6.7%) (*P* = .002). Animals in WBM group died predominantly during the first three days, whereas in the other group deaths occurred over time after surgery (Figure 1). Therefore, to evaluate the effect of encapsulated WBM on the regeneration pathway the remaining analyses were performed in the first 72 hours after 90% PH.

### 3.2. Expression of Genes Involved in Liver Regeneration

**Figure 2:**
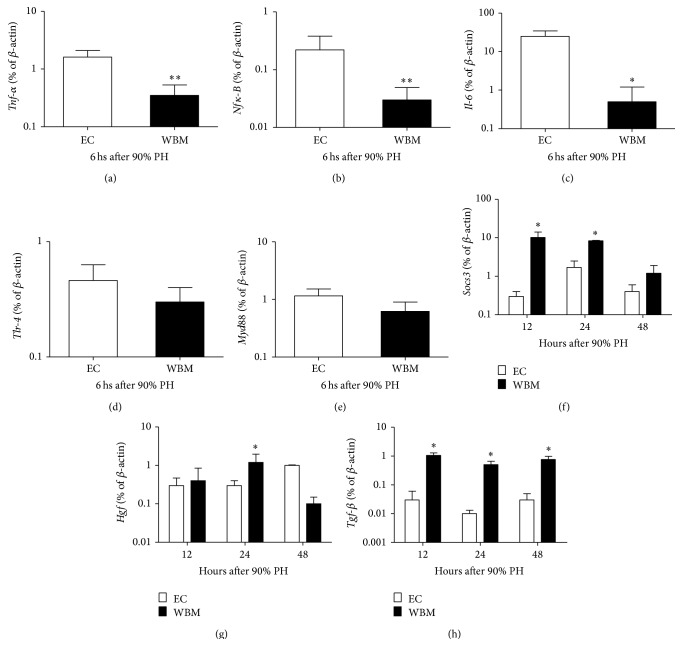
Liver gene expression after 90% partial hepatectomy (PH). Liver gene expression of* Tnf-α* (a),* Nfκ-B* (b),* Il-6* (c),* Tlr-4* (d), and* Myd88* (e) 6 hours after 90% partial hepatectomy and* Socs3* (f),* Hgf* (g), and* Tgf-β* (h) 12, 24, and 48 hours after 90% partial hepatectomy. WBM: whole bone marrow; EC: empty capsules. Values are expressed as means ± SD in log scale. Student's *t*-test, ^*∗*^ *P* < .05, ^*∗∗*^ *P* < .01.

First we assessed the expression levels of genes related to the early phase of liver regeneration. The expression of* Tnf-α* (*P* = .01) and* Nfκ-B* (*P* = .01) was markedly decreased in WBM group at 6 hours after 90% PH (Figures 2(a) and 2(b)). As a result, the expression of* Il-6* was also decreased (*P* = .04) in the WBM group compared to EC group (Figure 2(c)). Interestingly, LPS receptor (*Tlr-4*) and its mediator (*Myd88*) showed no differences in gene expression between groups 6 hours after 90% HP (Figures 2(d) and 2(e)).

We then analyzed genes related to the progress of liver regeneration. At 12, 24, and 48 hours after 90% PH other genes were also differently expressed between WBM and EC groups.* Socs3*, which inhibits signaling via* Il-6*, was increased in the WBM group at 12 and 24 hours after 90% PH (*P* ≤ .05, Figure 2(f)).* Hgf* was slightly increased in WBM only 24 hours after 90% PH (*P* = .04, Figure 2(g)), whereas the expression of* Tgf-β* was increased in WBM group in 12–48 hrs (*P* ≤ .03, Figure 2(h)).

### 3.3. Liver Regeneration Rate and Histology Analysis

**Figure 3:**
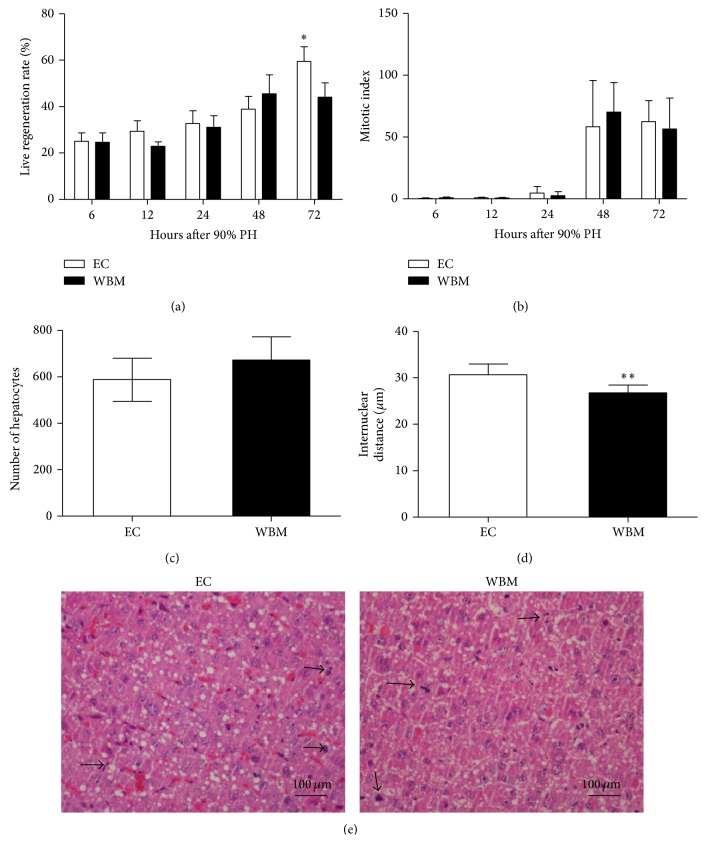
Liver regeneration rate after 90% partial hepatectomy (a). Mitotic index of hepatocytes after 90% partial hepatectomy (b). Number of hepatocytes (c) and internuclear distance (d) at 72 hours after partial hepatectomy. (e) Histology of mitotic hepatocytes (arrows) 72 hours after 90% partial hepatectomy; liver slides were stained with H-E. WBM: whole bone marrow; EC: empty capsules. Values are expressed as means ± SD. Student's *t*-test, ^*∗*^ *P* < .05, ^*∗∗*^ *P* < .01.

Interestingly, genes that promote liver regeneration were decreased in WBM group, whereas genes that halt hepatocyte division were increased. On the other hand, liver regeneration rate increased gradually after surgery, but without differences between groups at 6, 12, 24, or 48 hours. However, as shown in Figure 3(a), at 72 hours WBM group showed a lower regeneration rate compared to EC group (44% versus 59%, *P* = .003). Nevertheless, no differences were found in the number of mitotic cells in both groups (Figure 3(b)) and the number of hepatocytes at 72 hours after PH was also similar (Figure 3(c)). Surprisingly, the internuclear distance among hepatocytes was higher in EC group compared to WBM group at 72 hours (*P* = .003; Figure 3(d)), indicating that hepatocytes in WBM group were smaller than in EC group, resembling that of normal liver (data not shown). This could explain the lower regeneration rate, measured by changes in the remnant liver weight.

### 3.4. Mechanisms of Cell Death

**Figure 4:**
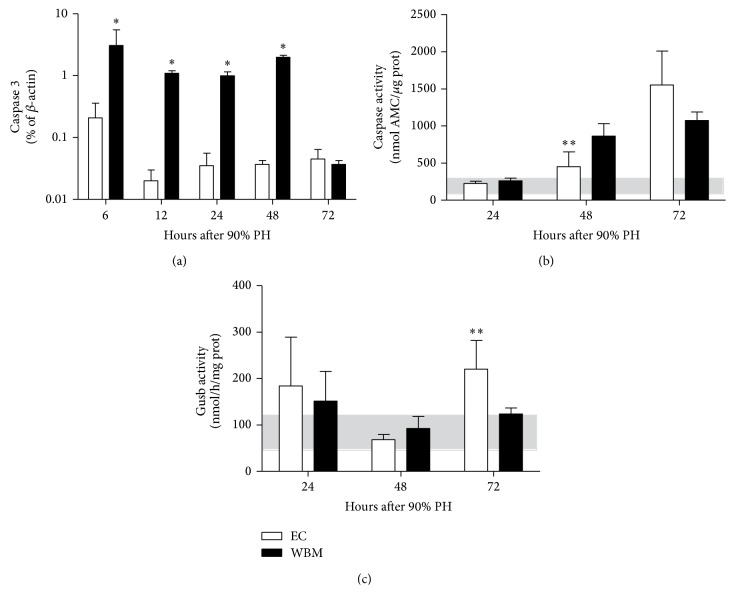
Mechanisms of cell death after 90% partial hepatectomy (PH). (a) Liver gene expression of* Caspase 3* at 6, 12, 24, 48, and 72 hours after 90% PH. (b) Caspase 3 activity and (c) lysosomal-*β*-glucuronidase (Gusb) activity at 24, 48, and 72 hours after 90% PH. Gray bar indicates normal values. WBM: whole bone marrow; EC: empty capsules. Values are expressed as means ± SD. Student's *t*-test, ^*∗*^ *P* < .05; ^*∗∗*^ *P* < .01.

Since no differences were found regarding cell proliferation, we then investigated if encapsulated WBM cells could lead to differential cell death. In order to assess possible mechanisms of cell death associated with our results, we quantified Caspase 3 as a measure of apoptosis and Gusb activity as an indicator of necrosis. We observed that WBM group had higher levels of* Casp3* at all times points (*P* < .05, Figure 4(a)), except at 72 hours where there was no difference between groups. Caspase 3 activity was also assessed in liver homogenates at 24, 48, and 72 hours. It was increased in WBM compared to EC group only at 48 hours (*P* = .013; Figure 4(b)), suggesting that cells from WBM group are dying by apoptosis. Interestingly when we evaluated Gusb activity at the same time point, WBM group presented less activity at 72 hours than EC group (*P* = .009; Figure 4(c)) suggesting that hepatocytes from EC group are dying by necrosis.

## 4. Discussion

In the present study we showed that encapsulated WBM cells increase ten-day survival in a model of 90% PH by acting very early in the regenerative process. At 6 hours after 90% PH the synthesis of inflammatory cytokines in the liver was reduced. Moreover, the expression of factors abrogating liver regeneration (such as* Tgfβ* and* Socs3*) was increased from 12 hours on, thus suggesting a decrease in the pace of liver regeneration through the secretion of paracrine factors. Our results corroborate the findings of Liu and Chang [13, 28, 29, 32], who showed that encapsulated WBM cells increased survival in rats after 90% PH.

The increase in 10-day survival rate from 6.7% in EC group to 63.6% in the treated group may not be directly comparable to other data in the literature. Indeed the survival rate after 90% PH is quite variable. It depends on many factors, including the surgeon's experience, the use of glucose, and the type of anesthesia [25]. In fact, some authors report 100% survival in one week [30] whereas others have 0% survival after 2 days [31], both using glucose supplementation as in the present study. Therefore, it is important to compare the differences between treated and untreated animals within the same research group, as all animals are submitted to the same surgeon, anesthetic protocol, and glucose administration. Also, there is no group without empty capsules (EC); therefore an influence of alginate itself in the survival curve cannot be ruled out. Yet, the results reported here can be compared to those of Liu and Chang [32] who reported 35% survival in empty capsules group in 10 days (and 100% in those treated with whole bone marrow cells). However, they showed an increase in the secretion of Hgf suggesting that it stimulated liver regeneration [32].

We evaluated the expression of inflammatory cytokines* Il-6*,* Tnf-α*, and* Nfκ-B* that are pivotal for the beginning of liver regeneration [33]. We observed that these cytokines were all decreased in WBM group at 6 hours after 90% PH. We then hypothesized that this reduction could be, at least in part, due to a decreased signaling by Kupffer cells. It is known that after partial hepatectomy Kupffer cells are overloaded with enteric antigens and that LPS-binding to Tlr-4 triggers the regenerative process [34]. However, no differences in expression of* Tlr-4* and its mediator* Myd88* were detected between groups. It is worth noticing that such differences may have occurred at earlier time points and therefore would not be detected by this study.

Consistent with this decrease in genes related to the promotion of early regenerative phase,* Hgf* was also not increased in WBM group, except at 24 hours after 90% PH. On the other hand, the expression of* Tgf-β*, an inhibitor of Hgf [35], was markedly increased in WBM group between 12 and 48 hours. In addition to that, the expression of* Socs3*, an important negative regulator of IL-6 that blocks Stat3 phosphorylation [36, 37], was also increased in WBM group. Taken together, these data suggest that encapsulated WBM cells are increasing survival by decreasing liver regeneration rate.

Nevertheless, the liver regeneration rate was similar in both groups until 48 hours. Only at 72 hours did WBM group show a decreased regeneration rate compared to EC group. Ninomiya et al. [22] suggested that the abrupt regenerative response after PH causes a derangement of the lobular architecture that is damaging to hepatocytes. In their work, the deceleration of liver regeneration increases survival rate after 90% PH. Accordingly, in the present study WBM group survival rate was 63% compared to 6.7% in EC group at 10 days after surgery.

It is important to stress that the rate of regeneration mentioned above is evaluated by the weight of the remaining liver. Thus, a more precise measure of regeneration rate would be mitotic index or hepatocyte number. However, when we evaluate these parameters we found no differences between the WBM group and EC group. Nevertheless, the internuclear distance was smaller in WBM group, suggesting that hepatocytes were smaller when compared with EC group. Therefore, these results point to the fact that hepatocytes of EC group are swelled and this may contribute to an increase in the reminiscent liver weight.

Cell swelling is an indication of hydropic degeneration, as observed by López et al. [37] in the 90% PH model. This led us to hypothesize that WBM group's hepatocytes are healthier than EC group's hepatocytes, maybe due to protective cell death. Both* Caspase 3* gene expression and activity were increased in the WBM group. Furthermore, Gusb activity, a marker of necrosis [38], was lower in WBM group. These results indicate that in the WBM group the predominant mechanism of cell death is apoptosis whereas in the EC group it is necrosis.

Apoptosis may be considered a controlled process to eliminate malfunctioning cells and results in apoptotic bodies that will be phagocyte by other cells [39]. Necrosis, on the other hand, is a traumatic cell death in which cells swell until the lysis and spread of intracellular components, which will trigger the immune response, leading to inflammation [39]. We observed that in both groups liver cells died as a consequence of injury; nonetheless in WBM group the death is cleaned and controlled.

It is worth noticing that donor and recipient animals were not related, as our experiments were performed in Wistar rats, which are outbred animals. However, as the cells are encapsulated in alginate beads no immune reaction against the cells is expected; that is the function of the capsules. The allograft model seems to be a better option as in a clinical setting one may not expect a patient in acute liver failure to be able to provide cells for transplantation or to wait for a match donor to be found.

In summary the results presented here show that encapsulated WBM cells increase survival in a model of 90% PH, reduce the expression of genes involved in liver regeneration, such as* Tnf*-*α*,* Nfκ-B*,* Il-6*, and* Hgf*, and increase those responsible for ending hepatocyte division, such as* Tgf-β* and* Socs3*. In addition to that, these cells favor apoptotic cell death and decrease necrosis, thus increasing long term survival. Although there is no definitive answer on how these cells exert their beneficial effects, a few hypotheses may be ruled out. There is no immunomodulatory effect of stem cells, as data on systemic cytokine levels did not differ between groups (Supplementary Figure 1 in Supplementary Material available online at http://dx.doi.org/10.1155/2016/4831524). Differences related to genes involved in liver regeneration were found but point to the opposite direction (as one would expect survival to be related to a faster regeneration). Also, no difference was found on cell proliferation. Unfortunately we were unable to retrieve enough RNA from recovered capsules in order to investigate what kind of changes happened in WBM cells, although preliminary data from an ongoing study from our group suggest that they may be compensating for some liver function, as well as which specific cell types are involved in this response.

## Supplementary Material

Serum IL-6 levels in rats after 90% partial hepatectomy (PH). ELISA for Interleukin 6 (R&D Biosystems) performed in serum collected at the time of euthanasia at 6 and 12 hours post-90% PH. EC (Empty Capsules), WBM (Whole Bone Marrow). Values are expressed as means ± SD. Student-t test, ^***^p<.0001 compared to control animals (without 90% PH).

## Figures and Tables

**Table 1:** TaqMan (Life Technologies, USA) ID assays for genes analyzed in this study.

Gene symbol	Assay ID
Act-*β*	Rn00667869_m1
*Hgf *	Rn00566673_m1
*Il-6 *	Rn01410330_m1
*Myd88 *	Rn01640049_m1
*Nfκ-B *	Rn01399583_m1
*Socs3 *	Rn00585674_m1
*Tgf-β*	Rn01475963_m1
*Tlr-4 *	Rn00569848_m1
*Tnf-α*	Rn00562055_m1
*Casp3 *	Rn00563902_m1

## Abbreviations

ALF: Acute liver failure
WBM: Whole bone marrow
EC: Empty capsules
PH: Partial hepatectomy
LPS: Lipopolysaccharides
*Tlr-4*: Toll like receptor 4
MYD88: Myeloid differentiation factor
*Tnf*: Tumor necrosis factor
*Il-6*: Interleukin-6
*Hgf*: Hepatocyte growth factor
*Tgf-β*: Transforming growth factor-beta
*Nfκ-B*: Nuclear factor kappa B
*Socs3*: Suppressor of cytokine signaling 3
Gusb: Lysosomal-*β*-glucuronidase
SD: Standard deviation.
