# Supplementary figures and images for: Encapsulated Whole Bone Marrow Cells Improve Survival in Wistar Rats after 90% Partial Hepatectomy
Source: Stem Cells Int. 2015 Nov 16;2016:4831524. doi: 10.1155/2016/4831524 (PMC4663362; doi:10.1155/2016/4831524)

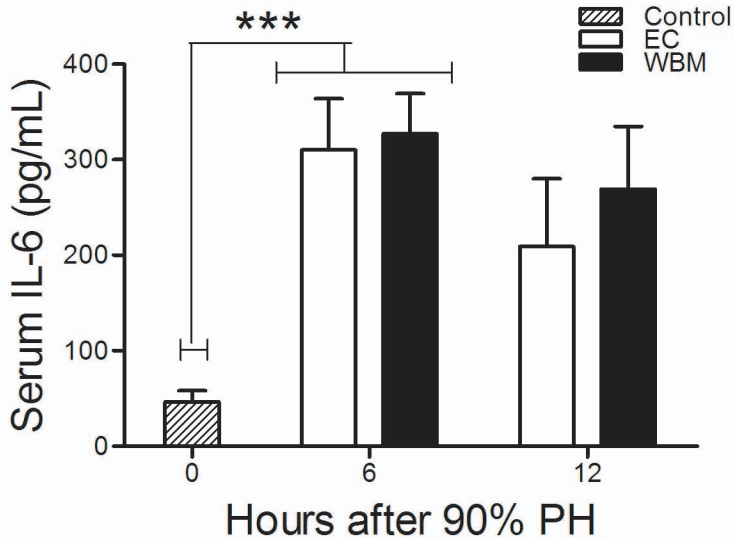

Supplement: Supplementary file 1 — Serum IL-6 levels in rats after 90% partial hepatectomy (PH). ELISA for Interleukin 6 (R&D Biosystems) performed in serum collected at the time of euthanasia at 6 and 12 hours post-90% PH. EC (Empty Capsules), WBM (Whole Bone Marrow). Values are expressed as means ± SD. Student-t test, ***p<.0001 compared to control animals (without 90% PH). [file 4831524.f1.pdf]
